# Atomic Insight into the Altered O^6^-Methylguanine-DNA Methyltransferase Protein Architecture in Gastric Cancer

**DOI:** 10.1371/journal.pone.0127741

**Published:** 2015-05-26

**Authors:** Naveed Anjum Chikan, Shoiab Bukhari, Nadeem Shabir, Asif Amin, Sheikh Shafi, Raies Ahmad Qadri, Trupti Navin Chandra Patel

**Affiliations:** 1 Division of Medical Biotechnology, School of Bioscience and Technology, VIT University, Vellore, Tamil Nadu, 632014, India; 2 Departments of Biotechnology, University of Kashmir, Srinagar, Kashmir, 190006, India; 3 Department of Animal Biotechnology, College of Veterinary Sciences, Anand Agricultural University, Anand, Gujarat, India, 388 001; 4 Department of Clinical Biochemistry, Sher-i- Kashmir Institute of Medical Sciences, Srinagar, Kashmir, 190011, India; Institut Jacques Monod, FRANCE

## Abstract

O^6^-methylguanine-DNA methyltransferase (MGMT) is one of the major DNA repair protein that counteracts the alkalyting agent-induced DNA damage by replacing O^6^-methylguanine (mutagenic lesion) back to guanine, eventually suppressing the mismatch errors and double strand crosslinks. Exonic alterations in the form of nucleotide polymorphism may result in altered protein structure that in turn can lead to the loss of function. In the present study, we focused on the population feared for high exposure to alkylating agents owing to their typical and specialized dietary habits. To this end, gastric cancer patients pooled out from the population were selected for the mutational screening of a specific error prone region of MGMT gene. We found that nearly 40% of the studied neoplastic samples harbored missense mutation at codon^151^ resulting into Serine to Isoleucine variation. This variation resulted in bringing about the structural disorder, subsequently ensuing into a major stoichiometric variance in recognition domain, substrate binding and selectivity loop of the active site of the MGMT protein, as observed under virtual microscope of molecular dynamics simulation (MDS). The atomic insight into MGMT protein by computational approach showed a significant change in the intra molecular hydrogen bond pattern, thus leading to the observed structural anomalies. To further examine the mutational implications on regulatory plugs of MGMT that holds the protein in a DNA-Binding position, a MDS based analysis was carried out on, all known physically interacting amino acids essentially clustered into groups based on their position and function. The results generated by physical-functional clustering of protein indicated that the identified mutation in the vicinity of the active site of MGMT protein causes the local and global destabilization of a protein by either eliminating the stabilizing salt bridges in cluster C3, C4, and C5 or by locally destabilizing the “protein stabilizing hing” mapped on C3-C4 cluster, preceding the active site.

## Introduction

Although declining, the malady of gastric cancer, according to GOLOBOCON 2012 is still the third leading cause of cancer deaths worldwide [1, 2]. In the pathogenesis of this disease, various genetic and molecular alterations take place leading to the malignant transformation of gastric mucosa [3]. This transformation is a multi step process that entails the abnormalities in important cellular functions such as DNA repair, adhesion, signal transduction, cell differentiation and others [4,5]. Alkylating carcinogens like N-Nitrosodimethylamine, Methyl Nitrosourea (NMU), N-methyl-N’-nitro-N-nitroguanidine etc. lead to formation of O^6^-Methylguanine, a DNA adduct whose presence leads to induction of mutations (G:C—A:T transition) and results in development of cancer [6–10]. *MGMT* is the enzyme responsible for the repairing O^6^-methylguanine adducts [11–13]. MGMT is a suicidal enzyme that removes a methyl group from the O^6^-position in guanine and transfers it to its own cystine residue at codon 145 in the protein, thus inactivating itself while repairing guanine [14]. Under the exposure of NMU, MGMT-defective mice have been seen to develop cancer [15], while as transgenic mice carrying extra copies of the foreign MGMT gene were less prone to the disease [16].The, genetic polymorphism of this enzyme has proven to be a potential risk factor for cancer [17–22]. This study thus focuses on mutational profiling of error prone region of Exon 5 of MGMT which encodes for the active site of the protein, viz active site surrounded by domains responsible for holding onto DNA [13]. The representative population of gastric cancer patients that has been selected for this study presents a unique cohort essentially being highly exposed to dietary alkylating agents [6, 23–28].

The use of *Insilico* techniques to understand the effect of polymorphism on protein structure and dynamics has been in practice and a plethora of work has been done in this regard [29–32]. The computer aided prediction methods using evolutionary and structure based prediction gives an insight into the damaging capability of the polymorphism [33]. The molecular dynamics can be used to observe the conformational changes the polymorphism can inflict in the protein. These conformational changes in the three dimensional structure of protein can affect the physiological affinities and various biochemical pathway interactions. To examine the effect of mutation at evolutionary as well as atomic level, Insilico predictions using different servers as well as MDS of the Wildtype (wt) and Mutant (Mu) MGMT protein was carried out. For MDS protein trajectories and atomic interaction analysis, gromacs inbuilt tools were used. Principle component analysis (PCA) was conducted to estimate the flexibility of both structures. Free energy landscapes (FEL) of native and Mu MGMT were also studied to comprehend the effect of mutation.

## Results and Discussion

**Fig 1:**
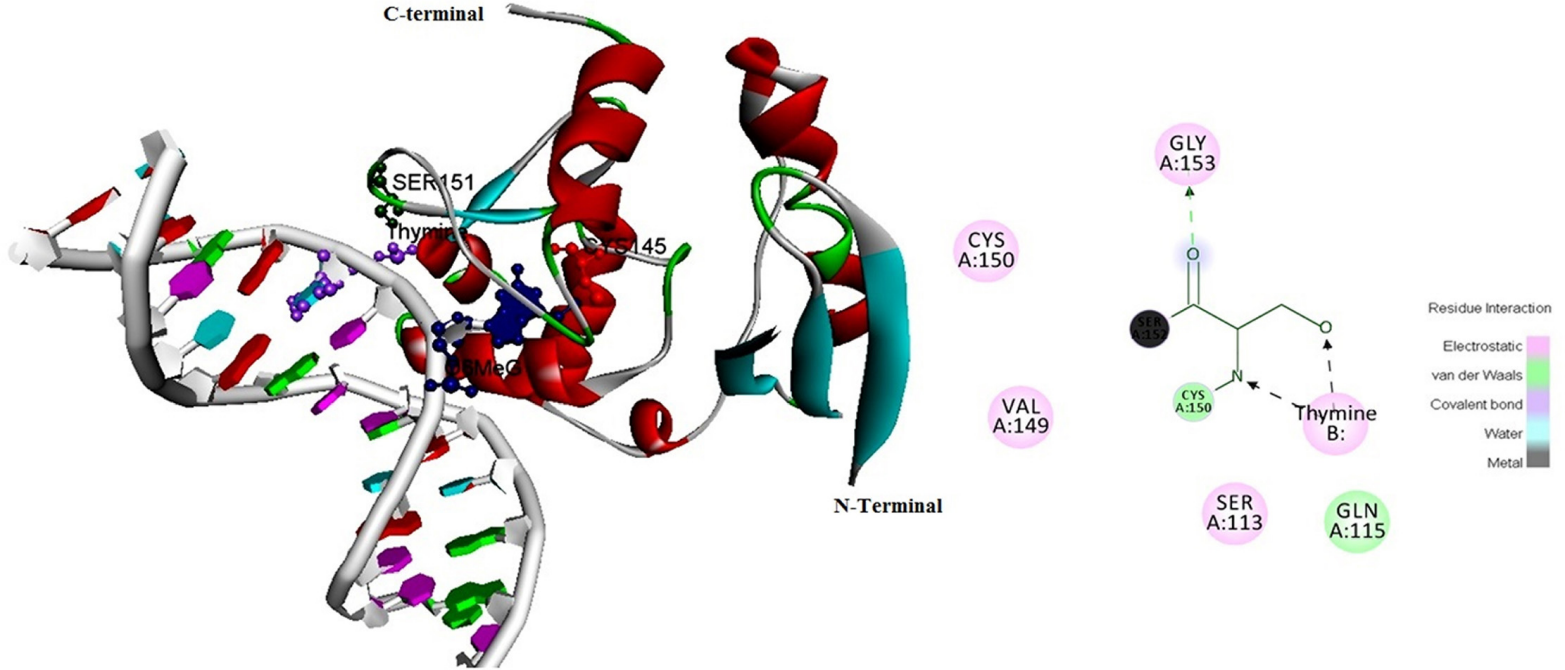
Representative picture of modeled wild type MGMT protein docked to minor groove of DNA depicts a stable interaction between Ser151 of enzyme with thymine base, attaining stability with the help of two hydrogen bonds shown in the figure as dotted arrows.

The Exon 5 segment of MGMT gene, was successfully amplified from all samples. Amplicons after sequencing showed a transversion mutation in codon 151AGC, the sequences of which have been submitted to GeneBank bearing accession numbers KM000795 and KM000796. The in silico tools to study the possible damaging effect of the mutation were selected meticulously, so as each factor is looked into and double checked by other tool which uses different algorithm. The details of the servers that are used in our study are described in S1 Table, where there algorithm, working and criteria for prediction is given. Selected server predicts the mutation to be damaging. The MDS simulation trajectories for 30ns run for wt and mutant protein were analyzed extensively using gromacs inbuilt tools. S1 Fig shows a nsSNP at codon 151 that leads to a missense mutation from Ser to Ile, otherwise in its wild-type form helps in Protein-DNA interactions [34–36]. As shown in Fig 1, wtMGMT (PDBID:1T39) SER 151, besides making normal electrostatic interaction with thymine also formed two hydrogen bonds with it via amide nitrogen.

**Fig 2:**
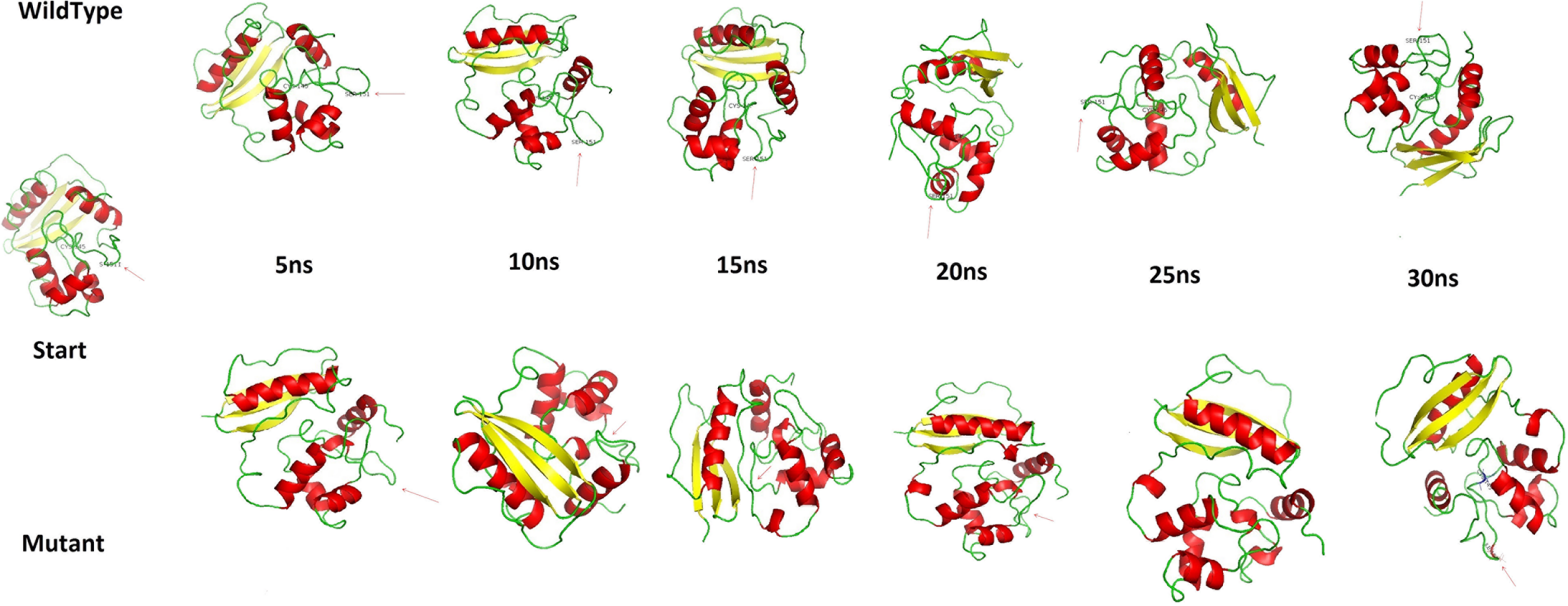
Illustrative representation of drastic conformational variability that a mutant structure (arrow specifies the site of an mutated residue) undergoes when compared wild type protein structure. The snapshots were retrieved at every 5 ns interval along the 30 ns simulation.

**Fig 3:**
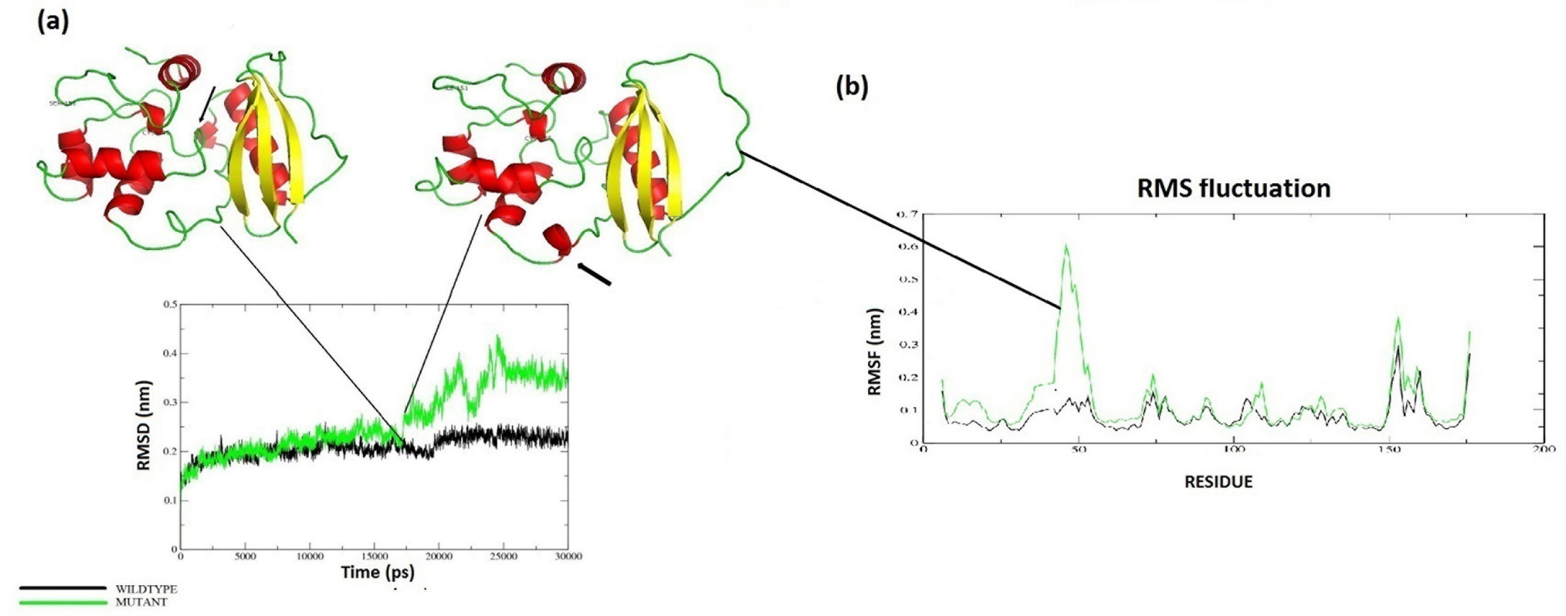
(a) Protein RMSDs for wt and Mu MGMT structures at 300 K. wt is shown in black and Mu in green. (Insets A and B) shows the relative structures at the point of RMSD jump. (b) MGMT residue RMSF along the MDS and the arrow pointing out to the region showing maximum fluctuation. (c) The RMSD vs. Atomic units. Plot showing highly unstable Mu curve in red.

Fig 2 shows the snapshots of both wt and Mu structures at different time intervals, stipulating the synopsis of the effect of mutation on structural dynamics of MGMT. From snap shots, the Mu structure other than revealing expanded conformation, also formed helical conformation at amino acid number 87 to 90, giving an idea that the mutation does not favor the structural compactness of the protein, which inturn leads to its compromised and aberrated conformation having a considerable structural shift that is pivotal in causing defunct protein function [37]. After visual analysis, g_rms tool was used to calculate the RMSD for protein atoms, using the starting structure as a reference. The mutant structure showed abrupt elevation in RMSD at around 17 ns. On observing the anomaly at the structure level, we found that helical and loop content of the mutant structure varied (Fig 3A). The RMSD from the average over time is referred as RMSF, g_rmsf was used to calculate the atomic standard deviation and on observation, the Mu structure showed higher flexibility. The RMSF of both structures showed a slight change at residue 151, but is varying considerably in a protein loop region of 27 to 53 (Fig 3B), which might be the resultant of an intermolecular long range tertiary interaction variation. In r_rmsf tool the option-oq was used to convert the RMSF value into B Factor values and implicit them on the average structure (blue representing the most stable and red most fluctuating). The comparative B factor projection (S2A Fig) on wt and Mu MGMT primarily indicates fluctuations variations within the average structure, giving us an insight into the change in the fluctuating pattern between the two structures. The coloring pattern is default ranging between blue to red. A significant change in fluctuation observed in Mu structure besides which the average secondary structure layout (S2B Fig) differed considerably which again implies that the Mu can be disadvantageous to DNA repair.

To analyze the shape of the protein at each given time, g_ gyrate tool was used, which calculates the Radius of Gyration of a group of atoms along the x-, y- and z-axis, as a function of time. Our results demonstrate the major deviation in Radii of gyration in Mu structure, passed after 17 ns run (S3 Fig). While as it is known that the MGMT structure does not vary to the great extent when compared to MGMT-bound-DNA structure, indicative of stable bound structure via close association of recognition residues (Ala126, Ala127, Ala129, Gly131 and Gly132), and Ser93, Thr95, Gln115, Asn123, and Ser151, interacting with the phosphate backbone of DNA[36] however since the radi of gyration was recorded to be increased due to the mutation and therefore suggesting the expanded overturned protein structure presumably awkwardly shifts the Arginine finger (intrahelical positioned Arg128) from its position, which is responsible for promoting the flipping of nucleotide into the MGMT active site, thus could impair the diligence needed for removing O^6^-methylguanine adduct from DNA

Further as we know that each amino acid has its own hydrophobicity-value, the original wild-type residue and newly introduced mutant residue differ in this property. To evaluate this, we used g_sas tool which computes hydrophobic, hydrophilic and total SASA of the protein over time. The mutant structure has greater SASA which correlates with our earlier finding of increased Rg in mutant structure (S4 Fig). To check the effect of the Mu on the MGMT structure docked with DNA PDB ID:1T39 [38], we used Discovery studio to color and compute the hydrophobicity according to kyte-dolittle scale (S5 Fig). The wt hydrophobicity and five residue running average hydrophobicity were -0.8 and 0.94 respectively, whereas the corresponding values for Mu residue were considerably higher at 4.5 and 2, thus showing that Mu residue is more hydrophobic than the wt residue. The indexed deviation in the values of mutant protein hydrophobicity relative to the wt protein could profoundly affect the stiochiochemistry of hydrogen bond formation between the enzyme and DNA, as is evident from S5 Fig. Subsequently, the unfavorable Enzyme-DNA docking can lead to non-responsiveness of the enzyme with respect to its cooperative functionality.

**Fig 4:**
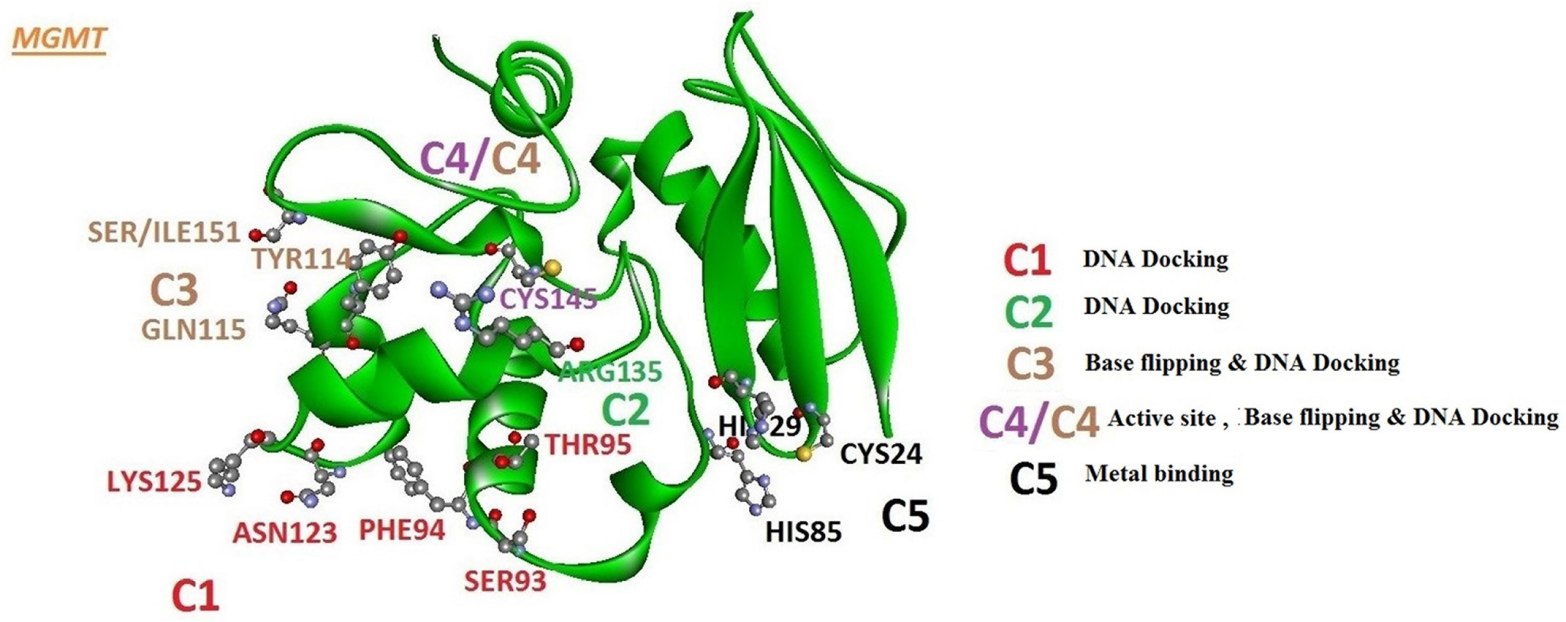
MGMT protein shown with the domains assembled into functionally important clusters that surround the active site.

**Fig 5:**
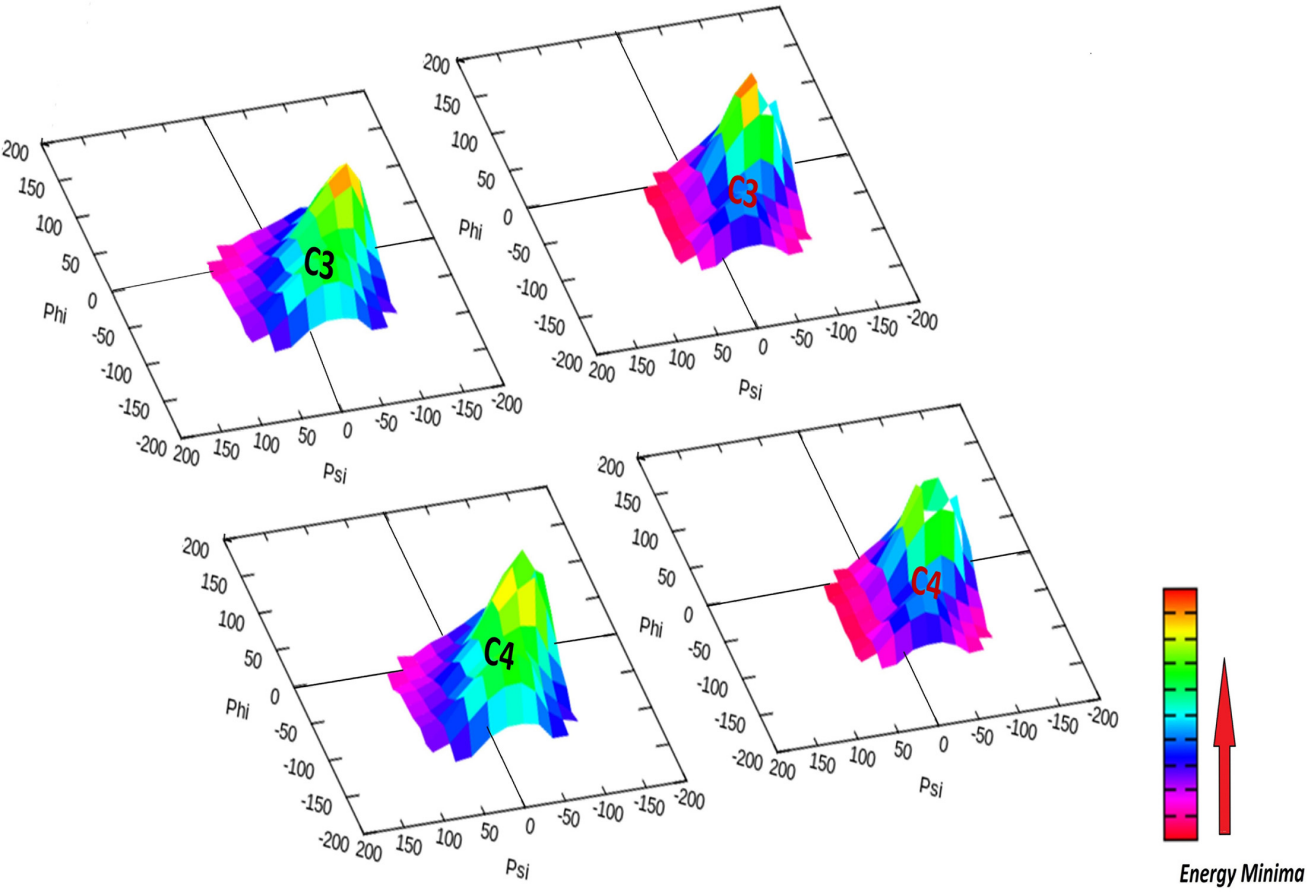
Three dimensional time dependent Phi/Psi distribution showing the change in the Gibbs free energy by the mutation in the Clusters 3 and 4 (black = wt, Red = Mu).

To further the understanding of mutation on protein dynamics, we divided important amino acids involved in physical interaction with DNA and Mg^+^ ion into clusters (Fig 4) depending upon their position and contributions in DNA docking, base flipping and DNA repair [36]. Cluster1 contained five amino-acids involving in DNA docking viz. SER93, PHE94, THR95, ASN123 and LYS125. Cluster 2 contained single amino acid ARG 135 also involved in DNA docking. Cluster 3 contained three amino-acids TYR114, GLN115 and SER151 where TYR 114 is involved in base flipping required for DNA repair and the other two have roles in DNA docking. Cluster 4, besides containing cluster 3 amino-acids, contained CYS145 which is an active site of MGMT, responsible for DNA repair. Cluster 5 consist three amino acids (CYS24, HIS29 and HIS85) all of which interact with Mg^+^ ion. g_rama tool was used to generate phi/psi dihedral combinations of selected clusters and was used to compute the angles as a function of time. Their contour plot was generated using energy minima to understand their respective mobility (S6 Fig). All the selected clusters were affected by the mutation from SER151 to ILE151. To understand the effect, particularly on cluster 3 and 4, the Psi /phi distributions pertaining to the labeled energy minima were plotted (Fig 5). The difference in the peak region of energy minima can be observed in the corresponding wt and Mu clusters, giving distinctive impression of possible imparity in DNA repair.

**Fig 6:**
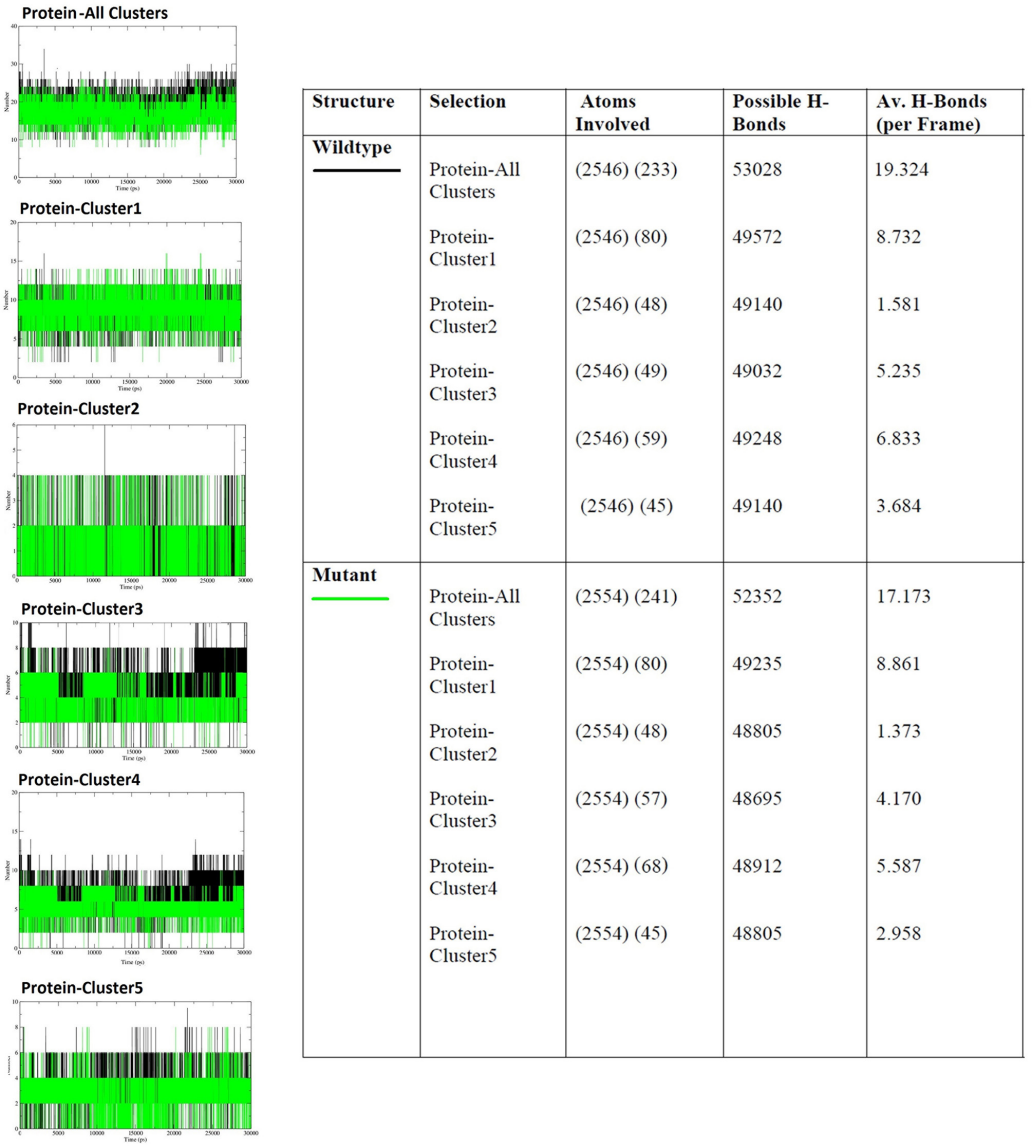
Intra protein hydrogen bond profile of wt and Mu MGMT protein over time at 300K.

For deeper understanding of the structural variation observed till now, we looked into intra hydrogen bond formation of the selected clusters using g_hband tool, the results of which have been shown in Fig 6. All the clusters selected for this analysis show the decrease in average number of hydrogen bonds per frame in mutant structure expect Cluster 1. The increase in the number of average hydrogen bonds per frame in Cluster 1 is slender in comparison to the variations we observe. The total decrease in the average hydrogen bond formation per frame is in co-relation with increased RMSF and Rg in mutant structure. The result generated by this analysis is conclusively entailing the anomaly observed till now with change in intra hydrogen bond pattern.

**Fig 7:**
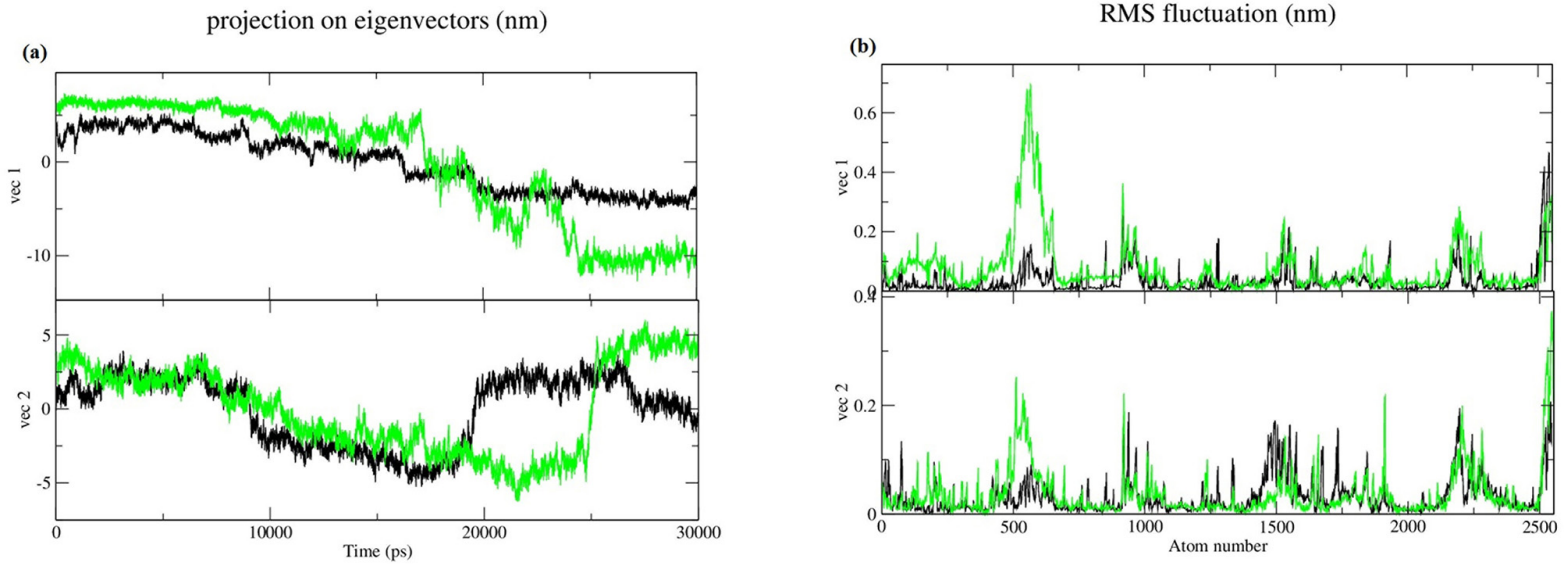
(a) The motion of principle EV overlaid sequentially. Black and green colour represents wt and Mu respectively (b) RMSF of all atoms of both vectors.

**Fig 8:**
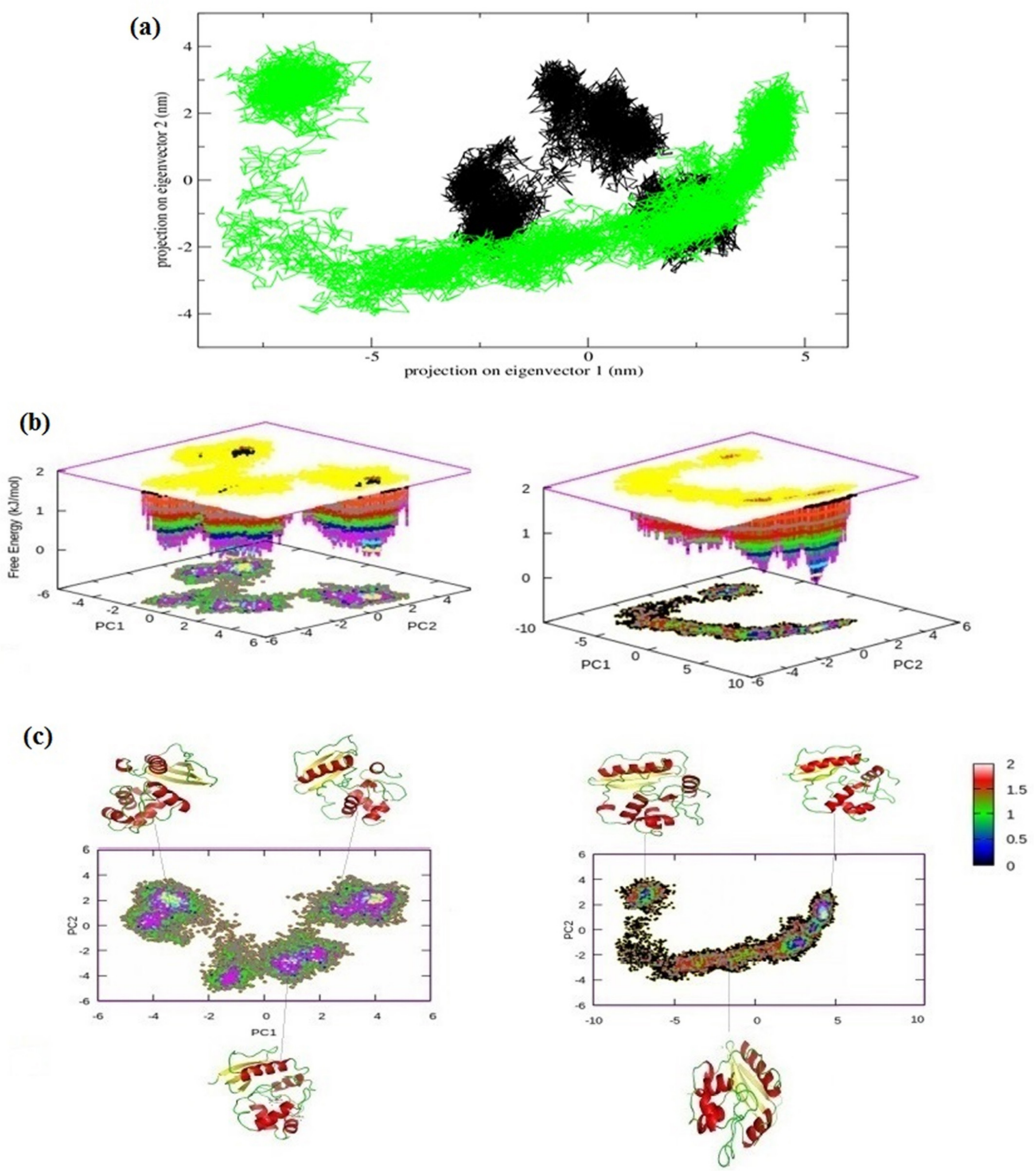
(a) Two dimensional representation of the motion of both structures along the first two principal eigenvectors, Black and green represent the wt and Mu MGMT respectively. (b) FEL of both the motions generated separately. (c) Separate two dimensional representations of PCA of both wt and Mu MGMT with the inset of three most stable structures at different point of time.

To understand the effect of this mutation on global correlated motions in atomic simulations, PCA, a mathematical technique that is efficient in characterizing the general folding and non-folding features of protein, was used. The technique identifies dominant motions in the protein by extracting principal modes involved in the motion involved in the molecule. The principal components of protein motion were computed as the eigenvectors (Ev) of the mass weighted covariance matrix of protein atoms. The calculation of these values was carried out using essential dynamics (ED) method according to standard protocol [39] available within the GROMACS software package. Two of the first eight Ev’s that account to more that 85% motion of overall system were selected for analysis, the projection over time and RMSF fluctuation of which is depicted in Fig 7. Both the Ev’s were combined into one single trajectory; the combination produced a common set of Principal Component (PC) eigenvectors for wt and Mu MGMT, making direct comparison possible among different systems. The trajectories were obtained using g-covar and g-anaeig of gromacs utilities. In Fig 8(A) the projections, PC 1 vs. PC 2, of both structures are projected (black wt/ red Mu), the cluster obtained from wt structure is stable, where as the projection of first two PC of mutant covers a large area. To further analyze the PC projections, their free energy surfaces were plotted (Fig 8B) which revealed that the stability of wt over the run is uniform over time as compared to Mu based on the energy minima basins formed by both. The structures with minimum energy were retrieved from the free energy land scape at different points of time. The structures on the right side of each projection in Fig 8(C) of PC are from the start of simulation to the left one from the near end of simulation. This analysis was crucial in elucidating the compromised free energy landscape of Mu structure, an observation that besides corroborating with our previous results, has conclusively implied a drastic conformational change in Mu structure.

## Conclusion

Incongruities of DNA repair and cancer etiology are synonyms in a way that it is the occurrence of the mutation that has been widely accepted as the basis of cancer. A mutation in a DNA repair protein that could impair its function (S7 Fig) can create pretumorigenic environment and can assist in cancer progression at any stage. MGMT being one of the important DNA repair protein has an essential role in maintaining genomic stability by removing O^6^Methyleguanine adducts. Thus, a significant genetic polymorphism in this protein will have an effect on cancer development and its progression. As none of the studies till date has reported mutational analysis of MGMT using MDS, it has primarily prompted us to look into the possibility of MGMT being mutated in a classified population where consumption of foods containing higher levels of N-nitroso compounds is common and gastric cancer is prevalent.

The use of molecular dynamics to study the effect of novel mutation at codon ^151^ has given us an insight into architecture of both the structures at an atomic level over a run of 30 ns period. The effect of the mutation was not only limited to its vicinity, but also impinged on overall structure including secondary elements at different locations of the protein. The structural transitions observed in secondary elements, promotes the collapse of structural architecture of Mu MGMT protein. The FEL obtained by quasiharmonic analysis (PCA) also concluded that the mutation considerably affects the stability of the MGMT over time, a factor that can hamper the normal stoichiometric fashion of DNA repair by MGMT.

The explored mutation in exon 5 appears to be associated with driver mutation, which seems to affect DNA/protein interaction, an important factor that could affect DNA docking, base flipping and ultimately repair mechanism, which if impaired, could also result in genome wide increase in O^6^ methyl guanine adducts leading to increased genomic instability.

## Materials and Methods

### Ethical statement

The protocols/experiments involving the use of human specimens were duly examined and approved by University Human Ethics Committee (UHEC), VIT University, Vellore (UHEC-VIT/2011).

### Patients and tissue collection

A total of 30 patients diagnosed with gastric carcinoma admitted to Sheri-Kashmir Institute of Medical sciences (SKIMS), Srinagar were considered for the study. Patients undergoing surgery as the primary treatment at different stages of the disease were recruited for the study with their consent. The characteristics of the studied patients are listed in S2 Table.

Tumor samples 5mm^3^ were excised from surgically resected specimens within the tumor mass, excluding the margin. Adjacent non-neoplastic samples of similar dimension were taken from the resection margin, approximately 10mm from the macroscopic tumor edge and subsequently confirmed as benign by routine histopathology at SKIMS. A total of 30 tumor and 30 normal tissue samples were collected, and stored at -80^°^C until analysis.

### DNA extraction and Polymerase Chain Reaction

DNA was extracted from 2mm^3^ tissue samples using DNA extraction kit (Hi Pura Mammalian Genomic DNA Isolation Kit—HiMedia). Concentration and quality of DNA was measured by routine spectrophotometeric analysis. Amplification of the Exon 5 regions of the MGMT exon, was carried out in gradient minicycler (Eppendorf) in a 25μl reaction mix containing 1 μl (400ng/μl) genomic DNA, DNA polymerase {1X PCR buffer (200mM Tris HCl, 200mM KCl, 50 mM, (NH4)2 SO4) supplied with 25mM Mgcl2,Fermentas}, Nuclease free water and 1 μl of forward (5’- GCCCGTGCAGGTACGGTCTT-3’) and reverse (5’- AGCTCCCGCTCCCTTGAGCC-3’) primers each. The annealing temperature was optimized at 65.5°C. To facilitate Polymerase Chain Reaction (PCR) product analysis for mutation, PCR product sequencing was carried out.

#### SNP Damage Prediction

The damage prediction of the polymorphisim was carried out using SIFT [40], Polyphen-2[41], PhD-SNP [42], MutPred [43], SNAP [44], SNPs & Go[45] and PoPMuSiC [46].

### Molecular dynamics simulation

MDS studies were performed by Gromacs 4.5.3 package [47]. For wt MGMT, the PDB structure 1QNT [48] was used as a starting structure for MDS. Accelrys Discovery Studio [49] was used to make the single point mutation on the wild type structure. Both, wt and Mu MGMT were applied with GROMOS96 43a1 force field and then placed in a model of a pre-equilibrated water bath and counter-ions were added to achieve a neutral box using the “genion” tool that comes along with gromacs package. Solvent molecules were restrained to the original position with a force constrain of 100Kcal/mol for 5000 steps before being subjected to energy minimization for 5000 iteration. For regulating the temperature inside the box, Berendsen temperature coupling method [50] was used. Electrostatic interactions were computed using the Particle Mesh Ewald method [51]. Ionizing state of the residues, pressure and other parameters were set in the standard range. Non-bonded pair list was updated after every 10 steps and conformations were stored every 2 pico seconds (ps). Position restraint simulation for 500 ps was implemented to allow solvent molecules to enter the cavity region of structure. Finally, system was subjected to MDS for 30 nano seconds (ns). Root mean Square Deviation (RMSD), Root Mean Square Fluctuation (RMSF), Solvent Accessible Surface Area (SASA), Radius of gyration (Rg) and PCA were carried out by using inbuilt gromacs tools. g_hbond was used to calculate the number of distinct hydrogen bonds formed by specific residues to other amino acids within the protein during simulations (NH bond). g_sham was used extensively to obtain free energy landscape. Graphs were plotted using Grace GUI toolkit 5.1.22 version while as free energy landscapes were plotted using gnuplot 4.6.0 version. All visualizations were carried out using Pymol, Ligplus, VMD [52] and graphs were plotted using Grace Program [53] and GNUPlot. Trajectories were analyzed using the inbuilt tool in the GROMACS distribution.

## Supporting Information

S1 Fig a) A representative chromatogram of MGMT exon 5 showing the single base pair, G>T at position 151 as indicated by an arrow in the neoplastic chromatogram. b) Alignment of exon 5 sequence that was amplified from neoplastic and non-neoplastic tissue (adjacent normal) with that of wild type (Reference-sequence acquired from NCBI) was translated and the SNP mapped was shown to change of Serine into Isoleucine.
(TIF)

S2 Fig (a) Average Tertiary structures colored according to Bfactor values (b) Average Secondary structure representation of both structures. (TIF)

S3 Fig (a) Radii of gyration of wt and Mu MGMT shown separately. (b) Rg of all atoms of wt and Mu MGMT versus time at 300K.
wt is represted by Black and Mu by Green.
(TIF)

S4 Fig Solvent Accessible Surface Area of wt (black) and Mu (green) MGMT over time at 300K. (TIF)

S5 Fig Variation In color (Surface of protein according to kyte-doolittle scale) at mutated region and the graphical representation of variation in kyte-doolittle scale in single amino acid and five average running hydrophobicity of both wt and Mu MGMT. (TIF)

S6 Fig Time dependent Ramachandran Contour plot of all the selected clusters over time, each line showing the transition of 1. (TIF)

S7 Fig Pictorial Representation of GC:AT Transition by impaired MGMT. (TIF)

S1 Table Polymorphism Prediction using different servers. (DOCX)

S2 Table Characteristics of Study Subjects. (DOCX)
