# Supplementary figures and images for: Atomic Insight into the Altered O6-Methylguanine-DNA Methyltransferase Protein Architecture in Gastric Cancer
Source: PLoS One. 2015 May 26;10(5):e0127741. doi: 10.1371/journal.pone.0127741 (PMC4444098; doi:10.1371/journal.pone.0127741)

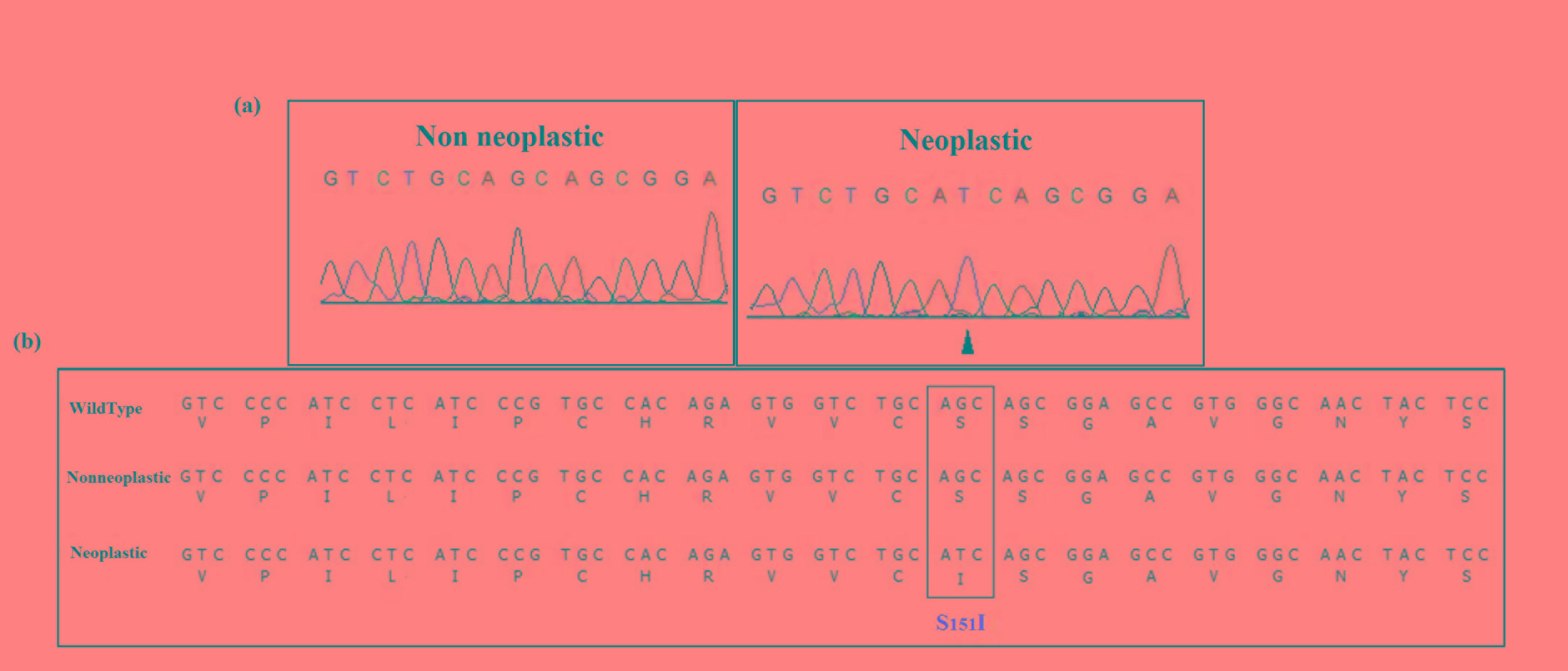

Supplement: S1 Fig — b) Alignment of exon 5 sequence that was amplified from neoplastic and non-neoplastic tissue (adjacent normal) with that of wild type (Reference-sequence acquired from NCBI) was translated and the SNP mapped was shown to change of Serine into Isoleucine. (TIF) [file pone.0127741.s001.tif]

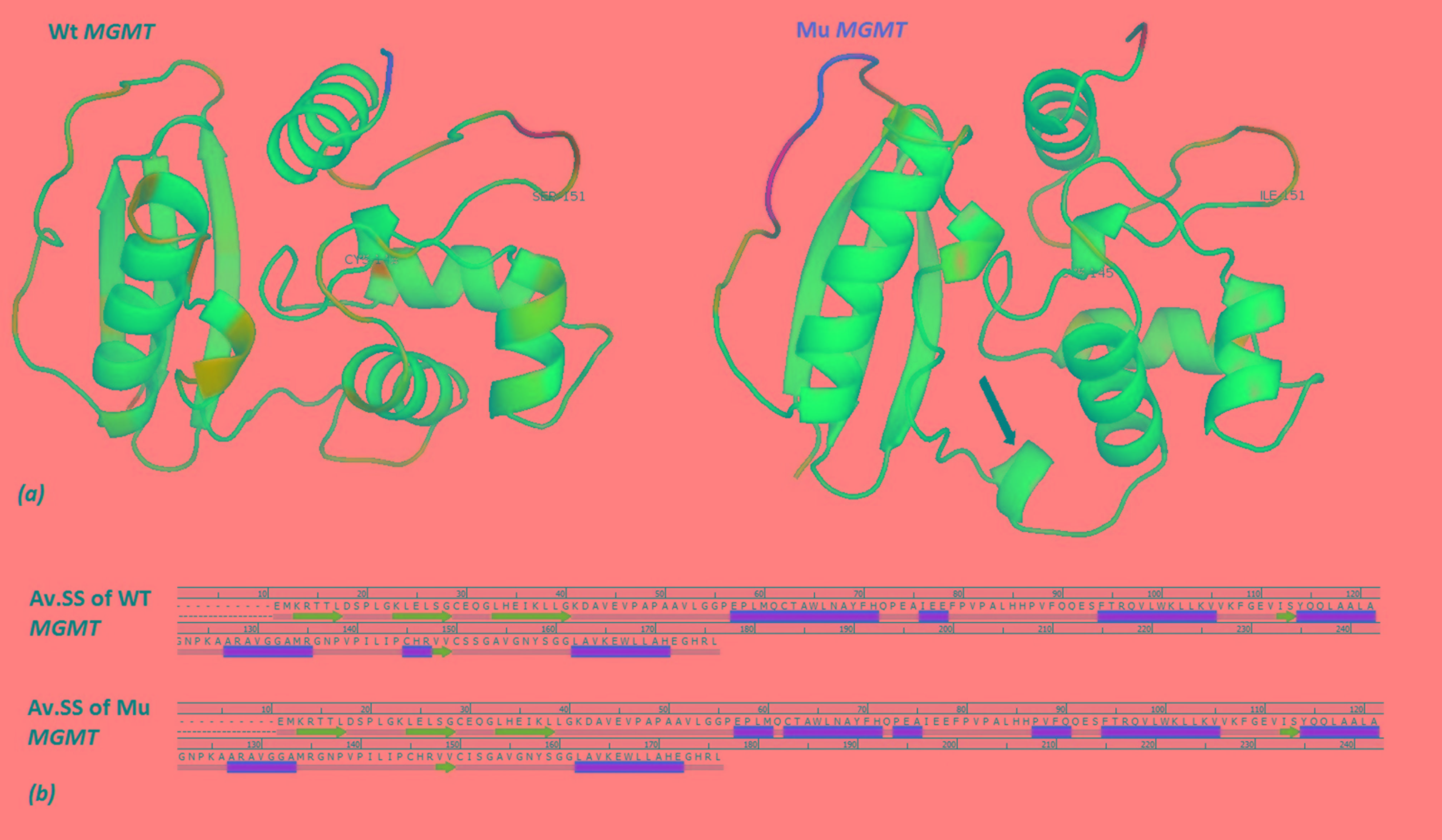

Supplement: S2 Fig — (TIF) [file pone.0127741.s002.tif]

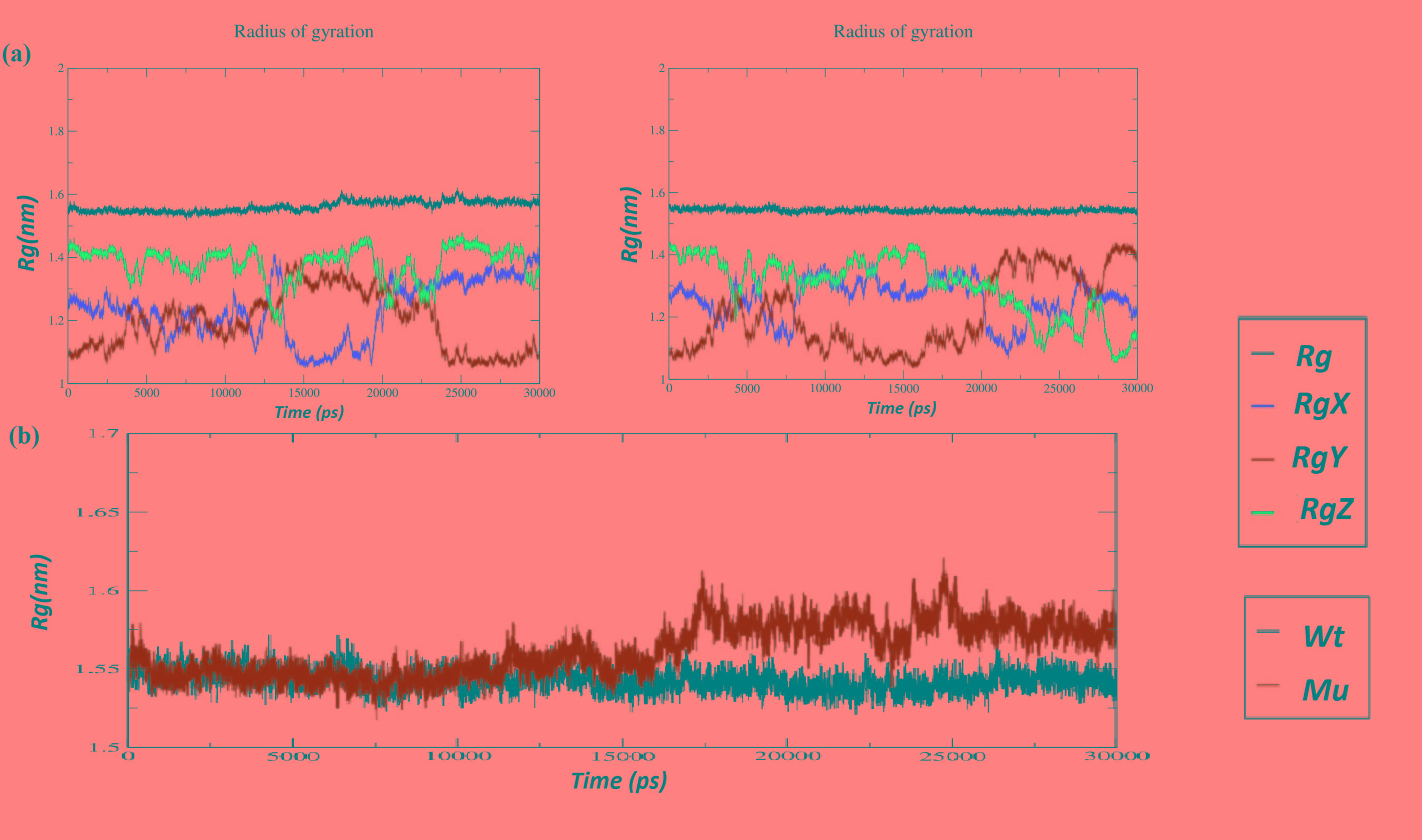

Supplement: S3 Fig — (b) Rg of all atoms of wt and Mu MGMT versus time at 300K. wt is represted by Black and Mu by Green. (TIF) [file pone.0127741.s003.TIF]

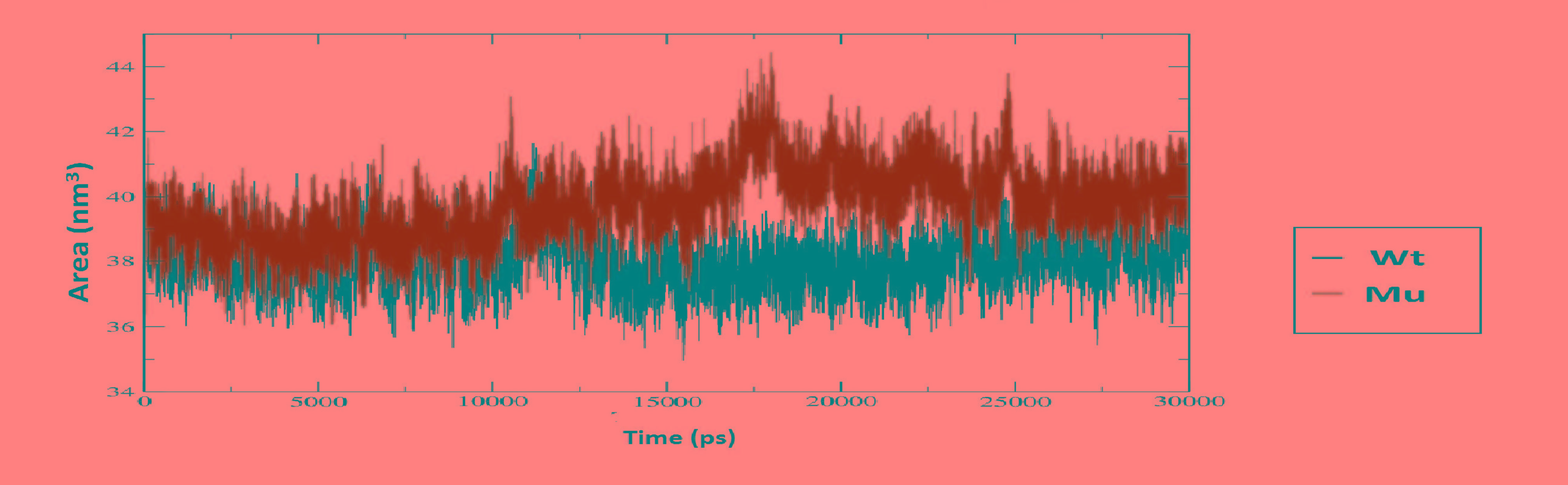

Supplement: S4 Fig — (TIF) [file pone.0127741.s004.TIF]

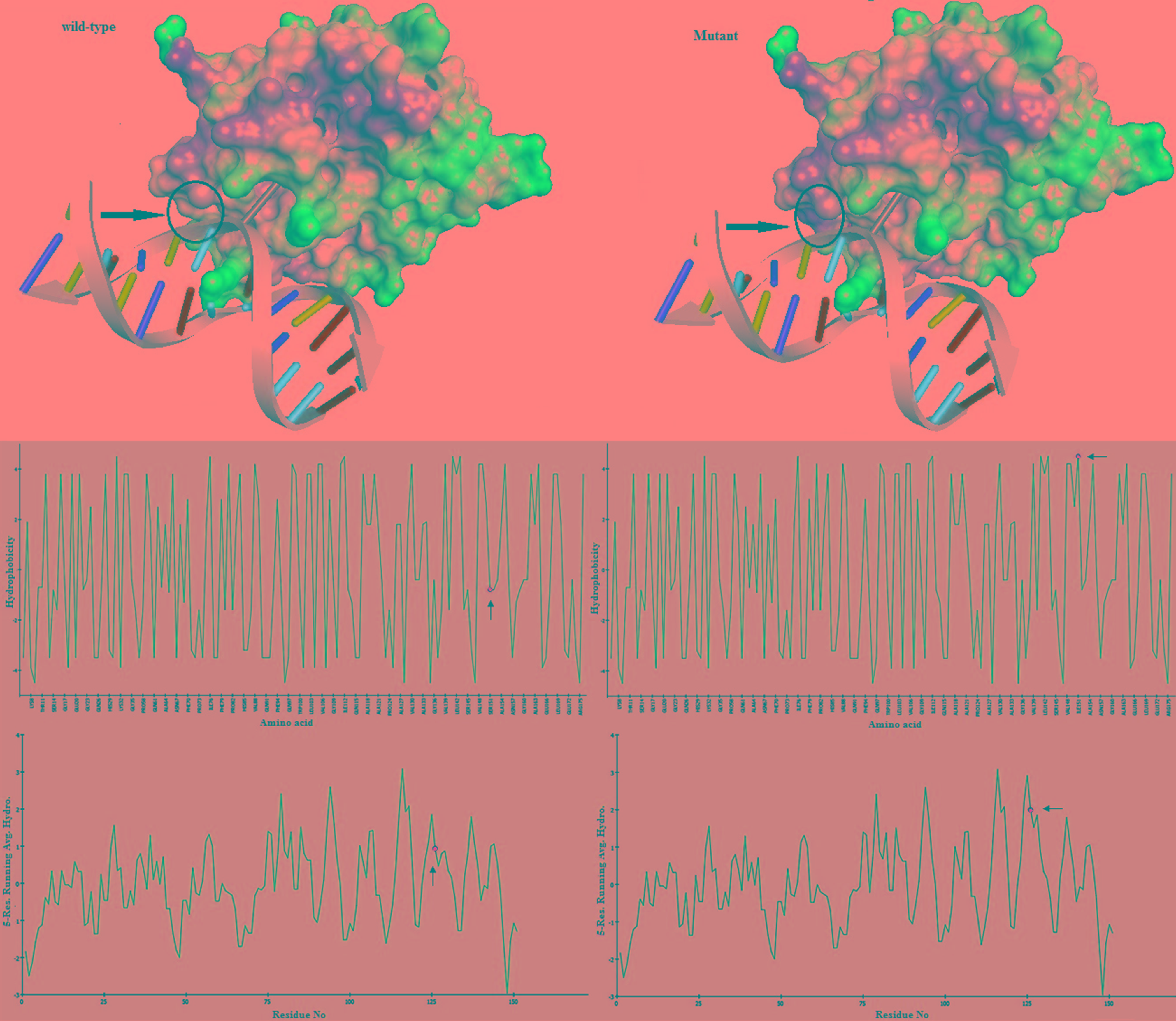

Supplement: S5 Fig — (TIF) [file pone.0127741.s005.TIF]

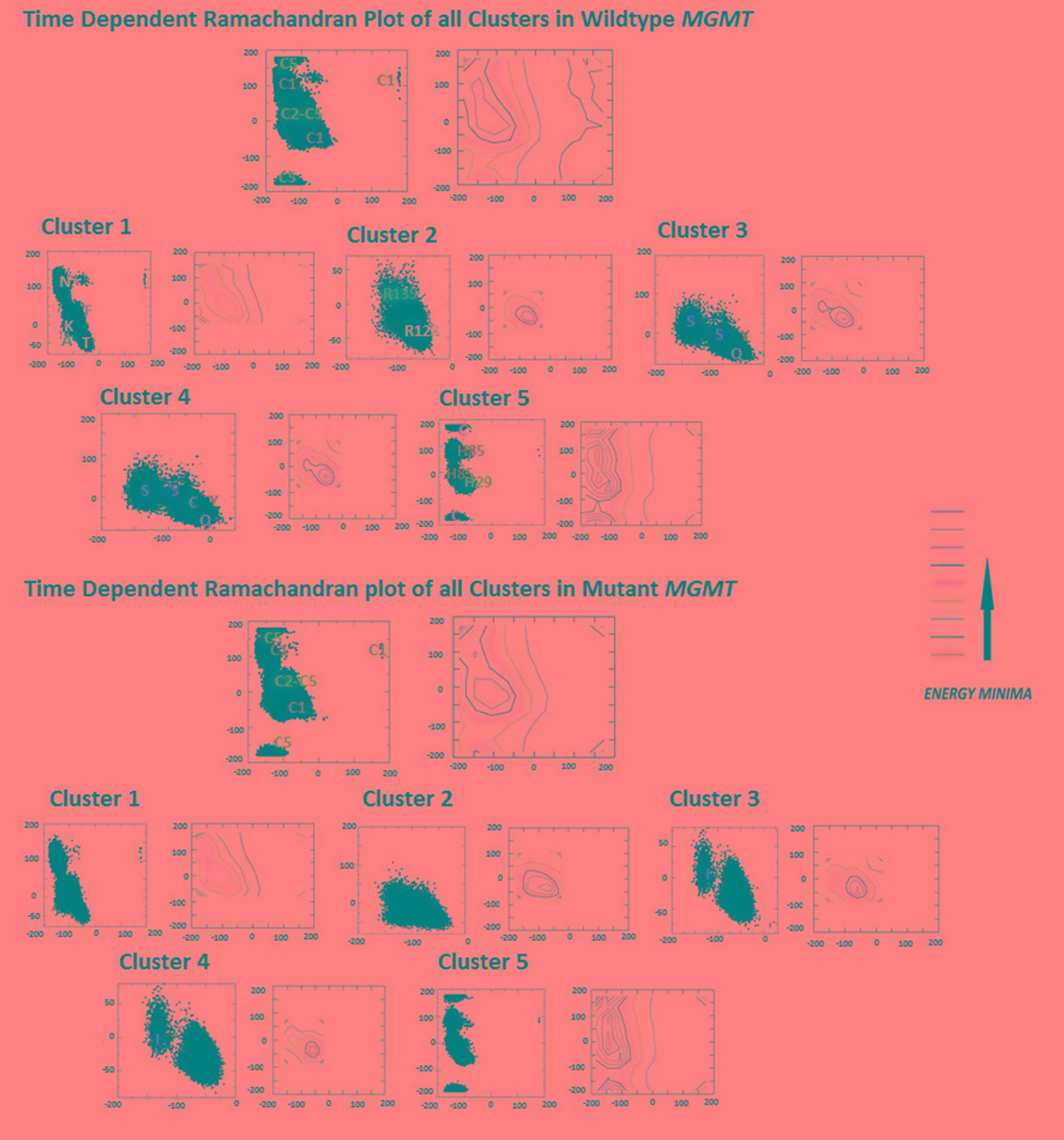

Supplement: S6 Fig — (TIF) [file pone.0127741.s006.TIF]

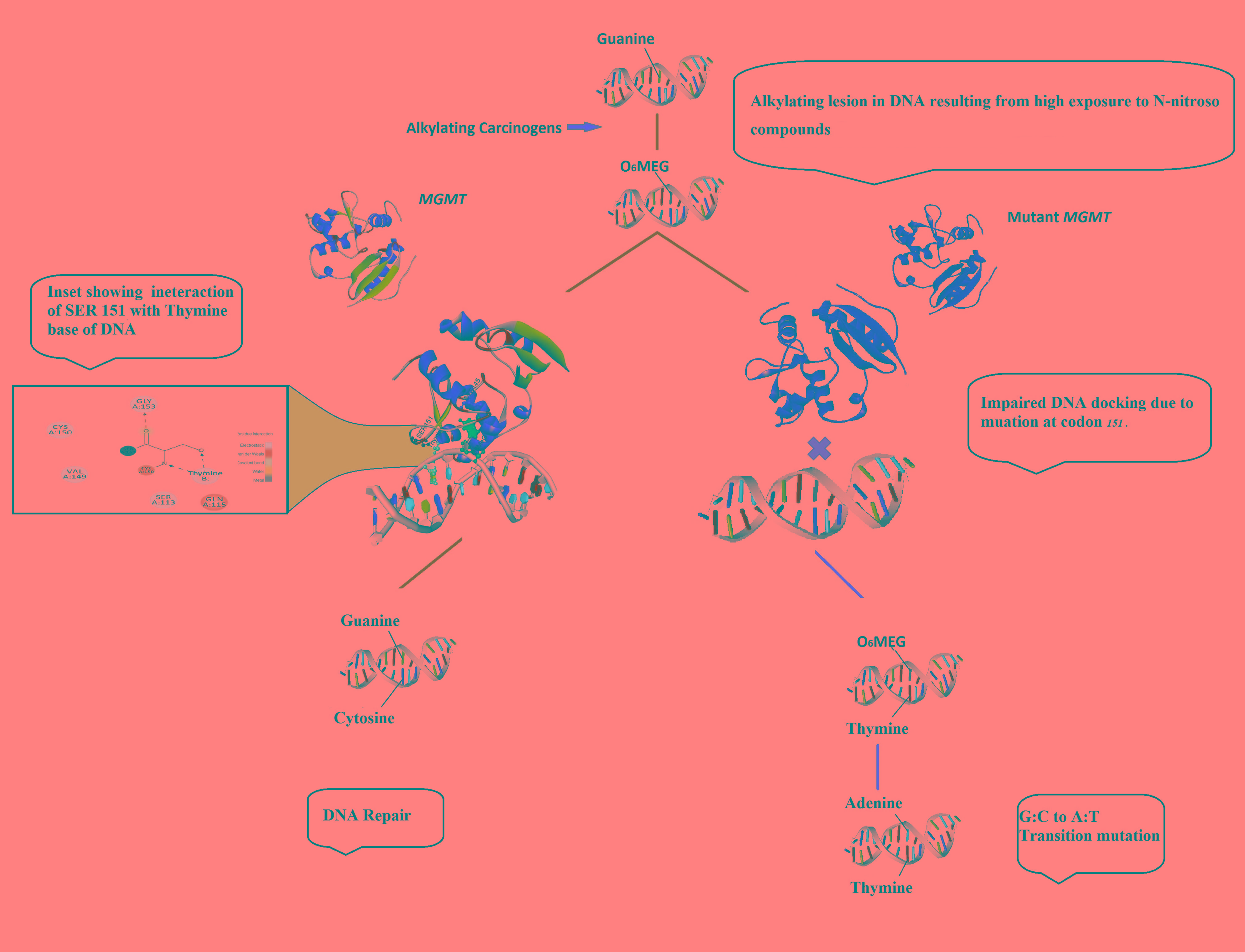

Supplement: S7 Fig — (TIF) [file pone.0127741.s007.TIF]
