# Supplementary material for: Atomic Insight into the Altered O6-Methylguanine-DNA Methyltransferase Protein Architecture in Gastric Cancer
Source: PLoS One. 2015 May 26;10(5):e0127741. doi: 10.1371/journal.pone.0127741 (PMC4444098; doi:10.1371/journal.pone.0127741)
Supplement: S1 Table — (DOCX) [file pone.0127741.s008.docx]

**S1 Table**

| **Method** | **Algorithm** | **Based on** | **Criteria** | **website** | **Prediction** |
| --- | --- | --- | --- | --- | --- |
| SIFT | Alignment  scores | Sequence alignment | 0 - 0.05  (Intolerant) | <http://sift.jcvi.org/www/SIFT_enst_submit.html> | **Damaging** |
| Phd-SNP | support vector  machine | Sequence & profile  information | Disease probability  (if P>0.5 mutation is predicted Disease) | <http://snps.path.uab.edu/phd-snp/phd-snp.html> | **Disease-related Polymorphism** |
| SNPs & Go | support vector  machine | Sequence, profile  & functional information | Disease probability  (if P>0.5 mutation is predicted Disease) | <http://snps-and-go.biocomp.unibo.it/snps-and-go/> | **Disease** |
| MutPred | random forest | SIFT &  Gain/loss of functions | Scores with g > 0.5 and p < 0.05 are referred to as actionable hypotheses. | <http://mutpred.mutdb.org/> | **Loss of catalytic residue at S151 (P = 0.0152)** |
| SNAP | neural network | Protein information | RI≥0  Expected accuracy ≥50 | <http://rostlab.org/services/snap/submit> | **Non-neutral** |
| Polyphen-2 | Bayesian  classification | Physical and comparative  considerations | Score≥ 0.5 | <http://genetics.bwh.harvard.edu/pph2/> | **Possibly Damaging** |
| PoPMuSiC | statistical potentials  and neural networks | Protein Structure stability | *ΔG values* | <http://babylone.ulb.ac.be/PoPV2a/index.php> | **Destabilizing** |
