# Supplementary material for: Atomic Insight into the Altered O6-Methylguanine-DNA Methyltransferase Protein Architecture in Gastric Cancer
Source: PLoS One. 2015 May 26;10(5):e0127741. doi: 10.1371/journal.pone.0127741 (PMC4444098; doi:10.1371/journal.pone.0127741)
Supplement: S2 Table — (DOCX) [file pone.0127741.s009.docx]

**S2 Table**

|  | Characteristics | No. (%) |
| --- | --- | --- |
| Gender | Male  Female | 22 (73.33)  08 (26.66) |
| Age (years) | 0-50  Above 50 | 09 (30)  21 (70) |
